# Maltose-Binding Protein (MBP), a Secretion-Enhancing Tag for Mammalian Protein Expression Systems

**DOI:** 10.1371/journal.pone.0152386

**Published:** 2016-03-30

**Authors:** Raphael Reuten, Denise Nikodemus, Maria B. Oliveira, Trushar R. Patel, Bent Brachvogel, Isabelle Breloy, Jörg Stetefeld, Manuel Koch

**Affiliations:** 1 Institute for Dental Research and Oral Musculoskeletal Biology, Medical Faculty, University of Cologne, Cologne, Germany; 2 Center for Biochemistry, Medical Faculty, University of Cologne, Cologne, Germany; 3 Department of Chemistry, University of Manitoba, 144 Dysart Road, Winnipeg, Manitoba RT3 2N2, Canada; 4 School of Biosciences, University of Birmingham, Edgbaston, B15 2TT, United Kingdom; 5 Experimental Neonatology, Department of Pediatrics and Adolescent Medicine, Medical Faculty, University of Cologne, Cologne, Germany; 6 Centre for Ecology, Evolution and Environmental Changes, Faculdade de Ciências, Universidade de Lisboa, Lisbon, Portugal; New England BioLabs, UNITED STATES

## Abstract

Recombinant proteins are commonly expressed in eukaryotic expression systems to ensure the formation of disulfide bridges and proper glycosylation. Although many proteins can be expressed easily, some proteins, sub-domains, and mutant protein versions can cause problems. Here, we investigated expression levels of recombinant extracellular, intracellular as well as transmembrane proteins tethered to different polypeptides in mammalian cell lines. Strikingly, fusion of proteins to the prokaryotic maltose-binding protein (MBP) generally enhanced protein production. MBP fusion proteins consistently exhibited the most robust increase in protein production in comparison to commonly used tags, e.g., the Fc, Glutathione S-transferase (GST), SlyD, and serum albumin (ser alb) tag. Moreover, proteins tethered to MBP revealed reduced numbers of dying cells upon transient transfection. In contrast to the Fc tag, MBP is a stable monomer and does not promote protein aggregation. Therefore, the MBP tag does not induce artificial dimerization of tethered proteins and provides a beneficial fusion tag for binding as well as cell adhesion studies. Using MBP we were able to secret a disease causing laminin β2 mutant protein (congenital nephrotic syndrome), which is normally retained in the endoplasmic reticulum. In summary, this study establishes MBP as a versatile expression tag for protein production in eukaryotic expression systems.

## Introduction

Recombinant expression of extracellular, intracellular as well as transmembrane proteins is essential for biochemical, structural, functional, and therapeutic studies. Since most proteins require numerous and specific post-translational modifications, eukaryotic expression systems are indispensable to produce recombinant proteins [1]. However, certain repeating folding domains containing fewer cysteines can also simply be produced in bacterial expression systems. Protein sections composed of Fibronectin type III repeats or von Willebrand factor type A domains, for example, can be easily expressed in large quantities in bacteria and purified natively [2]. Since some eukaryotic proteins produced in bacteria accumulate in inclusion bodies, many strategies for releasing and refolding have been developed. To avoid complicated refolding procedures after solubilization from inclusion bodies with 6 M guanidine-HCl or 8 M urea, eukaryotic proteins can also be fused to solubility promoting expression tags, e.g. the MBP or GST [3, 4] (for the abbreviation list see Table 1). The most successful expression and purification tag used in bacterial expression systems is MBP, which has been proposed to harbor chaperone activity [5]. In particular, aggregation-prone folding intermediates of recombinant proteins are prevented from self-associations when fused to MBP. This idea, to utilize for tags chaperones or other polypeptides that support folding, has been followed up in the past. For example, SlyD was identified as a protein harboring prolyl-isomerase as well as chaperone activities [6]. Gp41 from HIV-1 fused to two subunits of SlyD could be expressed in large amounts in *E*. *coli* compared to untagged protein [7]. Moreover, combination of SlyD with human FKBP12 further enhanced its production [8]. Knappe et al. [8] also suggested that the fusion of a catalytic prolyl- or disulfide-isomerase domain together with a protein-binding or chaperone domain would be a preferential combination.

**Table 1:** Abbreviation List.

BM40	signal peptide (osteonectin, SPARC)
CHO-K1	chinese hamster ovary cell line (subclone)
COS-7	kidney cells from a male adult african green monkey
DCC	deleted in colorectal cancer
DLS	dynamic light scattering
ECM	extracellular matrix
*E*.*coli*	*Escherichia coli*
eGFP	enhanced green fluorescent protein
ER	endoplasmatic reticulum
Gli1	glioma-associated oncogene
GST	glutathione S-transferase
HEK293	human embryonic kidney cell line
HSP	heat shock protein
Isom.	modified SlyD
LE	laminin-type epidermal growth factor-like domain
LN	laminin N-terminal domain
MBP	maltose binding protein
MEGF9	multiple epidermal growth factor-like domains protein 9
mFc	constant fragment (Fc part) from the mouse IgG protein
mFc-opt	codon optimized Fc part
MMP	matrix metalloproteinase
NTR	netrin-like domain
ser alb	serum albumin
SlyD	PPIase, FKBP type peptidyl-prolyl cis-trans isomerase

Although chaperones and folding promoting proteins are present in eukaryotic cells, it is tempting to speculate that co-expression of chaperones or different isomerases together with recombinant proteins would further support folding. So far only a few studies exist on protein folding in combination with expression tags in eukaryotic cells. The Fc fragment of immunoglobulin as well as serum albumin has been reported to enhance secretion of recombinant proteins in HEK293 cells [9]. The serum albumin tag enhances solubility and half-life of a fusion protein in plasma when it is administered to the blood circulation [10]. However, to date no report is available comparing different polypeptides that are used as expression tags for recombinant proteins in eukaryotic cells.

In this study we compared different fusion tags for the production of extracellular, intracellular, and transmembrane proteins in different eukaryotic cell lines. Surprisingly, the MBP tag consistently improved protein production levels when fused to proteins. Biochemical and biophysical analysis revealed that MBP expressed in eukaryotic cells is a monomeric protein and contains no N-glycosylations. Moreover, MBP reduced the number of dying cells during transient transfection. Interestingly, the MBP tag also allowed the secretion of mutant proteins or protein fragments which otherwise would be retained in the ER. Finally, we demonstrated that the MBP tag is beneficial for cell-attachment studies.

## Materials and Methods

### Vector and clone constructions

All pCEP-4 vectors contain the BM40 signal peptide sequence followed by a double Strep II purification tag, if not mentioned otherwise. The different expression tags were cloned via XbaI and BamHI into the cloning site (maltose-binding protein MBP: WP_040064394, aa: 29–392; mouse serum albumin: NP_033784, aa: 19–608; mouse immunoglobin gamma-1 heavy chain mFc: AAH03435, aa: 245–463; and SlyD AEH98223, aa: 1–154 followed by egggsgggsgggsgggs peptide—spacer; a kind gift from Franz X. Schmid, University of Bayreuth). The matrix proteins were cloned via NheI and BamHI sites adjacent to the expression tag into the different expression vectors (Netrin-1 delta: NP_032770, aa: 1–457; Netrin-4 delta: NM_021320, aa: 20–473; Netrin-4 full-length: NM_021320, aa: 20–628; β1LN-LEa1-4: XM_006514993, aa: 22–509; γ1LN-LEa1-4: NP_034813, aa: 43–442; eGFP: AB041904, aa: 1–239; β2LN-LEa1-4: NM_002292, aa: 33–467; β2LN-LEa1-4 R246Q: NM_002292, aa: 33–467 and R246Q; DCC ecto: NM_007831, aa: 26–1101; HSP90α: NM_005348, aa: 2–732; gli-1: NM_010296, aa: 603–1111; MMP14: NM_008608, aa: 24–582; MEGF9: NM_172694, aa: 32–600).

### Transfection

HEK293 cells were seeded at 90% confluency in a 24-well plate and allowed to adhere overnight. Next day, cells were transfected with different constructs in triplicates using FuGENE^®^ HD (Promega, Mannheim, Germany, Cat. no.: E2311). FuGENE^®^ HD was added to DMEM/F-12, GlutaMAX™ (Life Technologies, Darmstadt, Germany, Cat. no: 10565–018) at RT for 10 min and then incubated with different plasmid DNA for additional 20 min. The transfection reagent to plasmid DNA ratio was 1:3 (0.5 μg plasmid per well). The different transfection DNA mixtures were directly added to the respective wells and incubated for 24 h. On the next day the supernatant was discarded and fresh serum free medium was added for additional 24 h before the supernatant or cells were harvested for western blot analysis. To detect membrane bound proteins cells were lysed using the cOmplete Lysis-B EDTA-free Kit according to the manufacturing guidelines (Roche, Cat. no.: 04719948001).

### Western blot analysis

All constructs used in this study were fused to an N-terminal double Strep II tag. For detection of the constructs, Strep-Tactin®-HRP conjugate was used (IBA, Göttingen, Germany, Cat. no.: 2-1502-001). Samples were run in triplicates on a 10% SDS-PAGE gel and afterwards blotted for 1 h onto a nitrocellulose membrane. The membrane was blocked with 1 x PBS, 0.5% Tween 20 supplemented with 3% BSA for 1 h. Then the block solution was discarded and Strep-Tactin®-HRP detection solution (1:100000 dilution in 1 x PBS, 0.1% Tween 20) was directly added to the membrane for 1 h. The membrane was then washed twice with 1 x PBS, 0.1% Tween-20 and twice with 1 x PBS for 1 min before the membrane was developed using ECL™ (GE Healthcare Europe GmbH, Freiburg, Germany, Cat. no.: RPN2232). The intensity of the signals (arbitrary signal) was analyzed using ImageJ.

### Dynamic light scattering

The Nano-S Dynamic Light Scattering system (Malvern Instruments Ltd, Malvern, UK) equipped with a 633 nm laser and a fixed scattering angle (173°) was employed to analyze purity of MBP and MBP-netrin-4 delta protein as described previously [11, 12]. Traditional DLS analysis provides the translational diffusion coefficient that was converted to Stokes radius or hydrodynamic radius via the Stokes−Einstein relationship using the DTS software (Malvern Instruments Ltd., Malvern, UK) supplied with the instrument. Both proteins were allowed to equilibrate for 4 min at 20°C prior to DLS measurements, after which multiple records of the DLS profile were collected for data analysis. Purity of MBP-netrin-4 delta (delta: missing the C-terminal NTR domain) was studied over the range of concentrations from 3.28 to 1.7 mg/ml whereas MBP alone was studied from 1.80 to 1.0 mg/ml in 1 x TBS buffer.

### Apoptosis analysis by Annexin A5 staining after transient transfection

Supernatant of transfected cells was collected into a 15 ml Falcon tube and cells were washed once with 2 ml of 1 x PBS. The wash fractions were added to the 15 ml Falcon tube with the respective supernatants. Then the adherent cells were treated with 0.05% (w/v) trypsin/ 0.02% EDTA (w/v) in 1 x PBS (Biochrom AG, Berlin, Germany, Cat. no.: L2153) at 37°C for 5 min. The trypsinized cells were also placed to the 15 ml Falcon tube containing the respective supernatants as well as the wash fractions and centrifuged with 300 x g at 4°C for 5 min. The cells from the different transfection approaches were washed once with ice-cold 1 x PBS containing 10% FCS. The cells were further washed in ice-prechilled binding buffer (10 mM HEPES, 140 mM NaCl, 2.5 mM CaCl_2_ in H_2_O pH 7.4). Afterwards 50 μl of staining solution (AnxA5-Dye490 1:60 diluted in binding buffer) was added to each fraction and incubated in the dark at RT for 15 min as described previously [13]. Staining was stopped by putting the samples on ice and addition of 150 μl of ice-cold binding buffer. Before measuring the different samples propidium iodide was added to each tube for 2 min. The samples were analyzed by flow cytometry (FACSCanto2, Becton Dickinson).

### Detection of eGFP

50 μl cell supernatant were transferred into 96 wells (Corning Costar black plates with clear bottom) and followed by the detection of eGFP emission signal using the Tecan Infinite M1000 reader at 509 nm (arbitrary signal).

### Cell adhesion assay

The wells of a 96-well plate were coated overnight with serial dilutions (0–50 μg/mL) of protein at 4°C. Trypsinized B16-F1 cells were resuspended in serum-free DMEM/F-12 (supplemented with 2 mM MgCl_2_ and 1 mM MnCl_2_) and 5 x 10^4^ cells/well were seeded in triplicates. Cells were allowed to adhere to the various substrates at 37°C for 30 min. After carefully washing away non-adherent cells once with 1 x PBS, adherent cells were fixed with 1% glutaraldehyde in 1 x PBS for 15 min at room temperature before staining for 25 min with 0.1% crystal violet in H_2_O. Adherent cells were quantified by releasing the dye from the cells with 0.2% Triton X-100. The absorbance was measured in a spectrophotometer (Tecan) at 570 nm. A blank value corresponding to BSA coated wells was subtracted.

### Glycan analysis

N-Linked glycans were released from the protein by PNGaseF digestion (BioLabs, 250 U) in 50 mM NH_4_HCO_3_ (pH 8.5) at 37°C for 16 h. After drying by vacuum rotation, the glycans were solubilized in 0.1% trifluoroacetic acid and separated from residual proteins by C18 extraction (BondElutC18, Waters). The O-glycan chains were released from protein by incubating the glycoproteins with 0.5 M NaBH_4_ in 50 mM NaOH at 50°C for 18 h (reductive β-elimination). The reaction was stopped by adding 1 μl acetic acid into each sample. Salt was removed with 50μl Dowex 50WX8 (BioRad) in a batch procedure. Excessive borate was codistilled as methylester in a stream of nitrogen by adding several 0.1 ml aliquots of 1% acetic acid in methanol. Permethylation of the glycan chains, released by reductive β-elimination, was performed as described by Ciucanu and Kerek [14]. Glycan analysis of the permethylated oligosaccharides was performed using MALDI-TOF/TOF (UltrafleXtreme, Bruker) with a Smartbeam laser and positive ion detection in the reflectron mode. As matrix 2.5 dihydroxybenzoic acid (Bruker) was used (40 mg/ml in acetonitrile/0.1% TFA 2:1) on a stainless steel target. The spectra were analyzed with the software *Flex Analysis*. Glycopeptide analysis was done by collision-induced dissociation-electrospray (CID) mass spectrometry: Recombinant proteins in solution were reduced and alkylated before digestion with trypsin or endoproteinase GluC at 37°C overnight. ESI-MS/MS analysis of the tryptic peptides was run on an ESI iontrap, the HCT ultra ETDII PTMDiscovery-System (Bruker) coupled with an online easy-nano-LC system (Proxeon). The sample was separated on an analytical C18 column (75 μm x 10 cm) using gradient runs from 0–35% acetonitrile in 0.1% TFA during 30 min. Ions were scanned with 8100 amu/sec in a range from m/z 300 to 2500 in MS mode and m/z 200 to 3000 in MS/MS mode. MS/MS spectra were generated by CID fragmentation.

### Homology modeling

A homology modeling approach was used to generate 3D structures of the MBP protein from *P*. *aerophilum*. The crystal structure coordinates of *P*. *furiosus* (PDBID: 1ELJ) were used as template for model building: The *P*. *aerophilum* MBP protein has 52% sequence identity and about 78% sequence similarity to *P*. *furiosus*. The DeepView tool of the SWISS-MODEL [15, 16] web server was employed to align the sequences in order to generate the template for the model building software Modeller [17].

## Results

**Fig 1:**
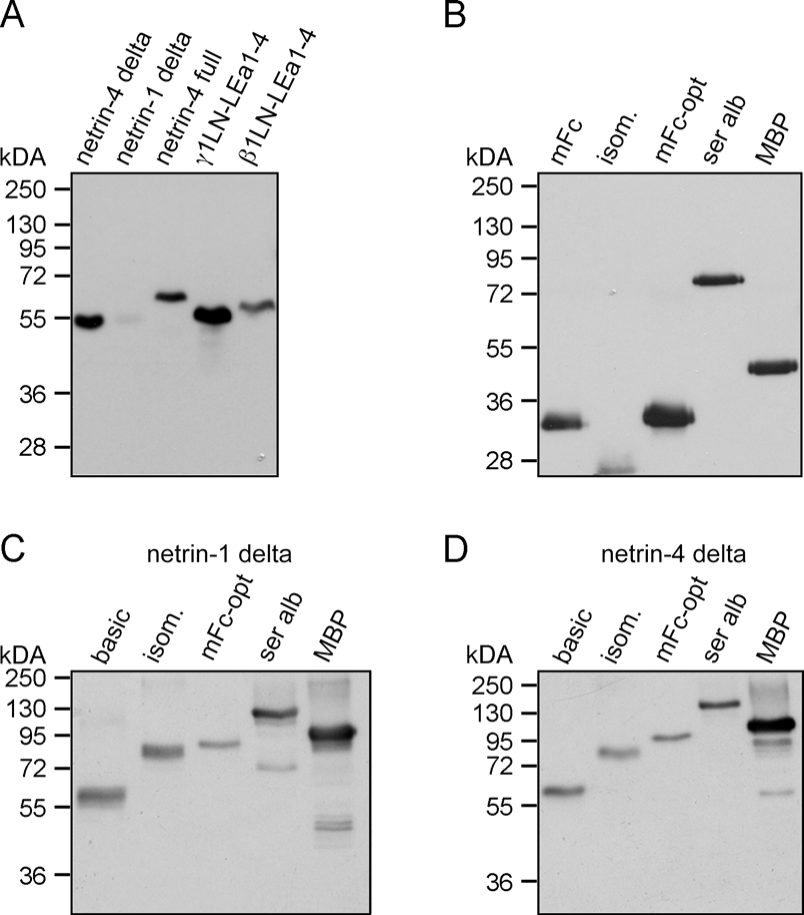
Expression analysis in HEK293 cells of commonly used expression tags. (A-D) The expression levels were compared by western blot analysis detecting the double Strep II tag, which is present as an N-terminal tag on all proteins. (A) Different short arm laminin chains and netrins were transiently expressed and analyzed by western blot. (B) Different expression tags were transiently expressed and visualized by western blot. (C) Netrin-1 delta was fused to different expression tags. (D) Expression of netrin-4 delta in different vector systems was analyzed by western blot analysis. (Isom.: modified SlyD; mFc: Fc part from the mouse IgG protein; mFc-opt: codon optimized Fc part from the mouse IgG protein; ser alb: serum albumin; MBP: maltose binding protein; basic: without fusion protein).

**Fig 2:**
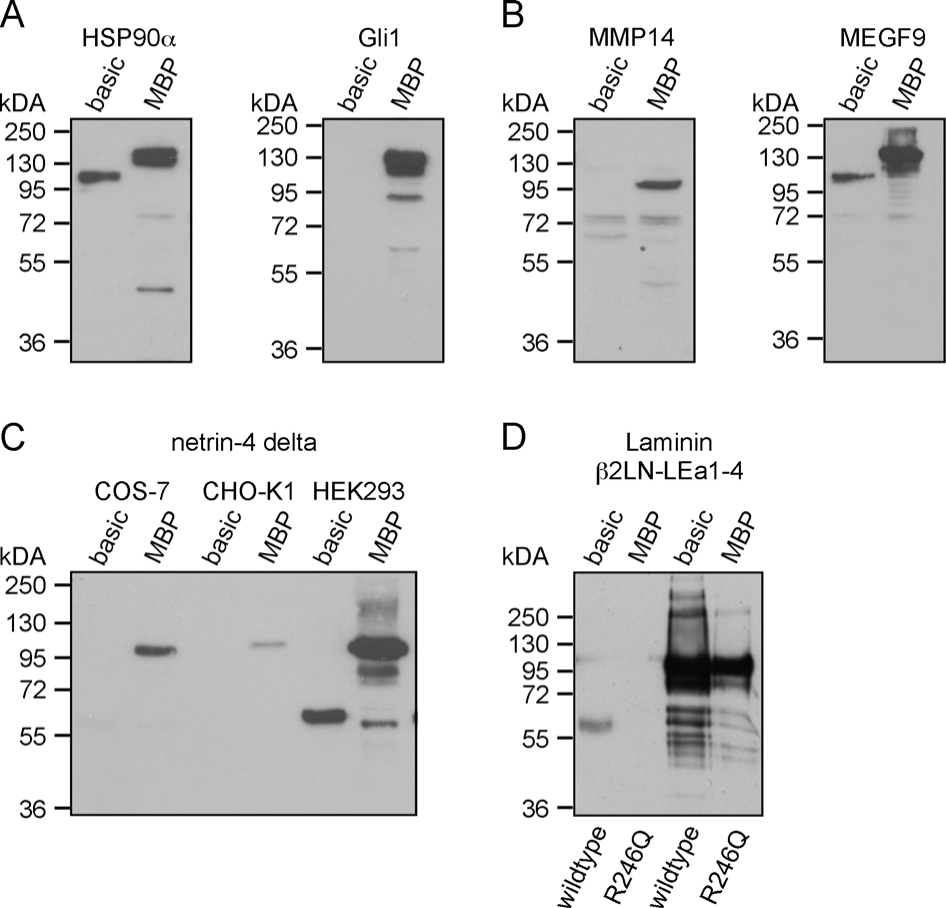
Global application of the MBP tag as an expression enhancing tag. (A) Protein expression levels in HEK293 cells of intracellular proteins (HSP90α and Gli1) with and without MBP. (B) Western blot analysis of transmembrane proteins (MEGF9 and MMP14) cloned into the basic and the MBP vector system. (C) Comparison of the expression levels of netrin-4 delta in the basic and the MBP vector transfected into COS-7, CHO-K1, and HEK293 cells. (D) The N-terminal laminin β2LN-LEa1-4 fragment and the respective R246Q mutant version causing congenital nephrotic syndrome were expressed and western blot analysis was performed.

The production of recombinant proteins in large quantities with excellent quality is still a bottleneck for performing biochemical, structural, and functional studies. In contrast to the production of antibodies in COS-7 or HEK293 cells (up to 5 g/L), the expression levels of different extracellular, intracellular as well as transmembrane proteins are much lower. Recovery between 0.5 and 20 mg/L of conditioned medium can be achieved in HEK293 (using triple flasks) with an episomal expression system. In our laboratory we use an N-terminal double Strep II tag for recombinant protein purification (S1A and S1D Fig). This purification tag offers several benefits due to the possibility of supplementing the cell medium with serum and washing the purification column with high salt buffer (1 M NaCl) without abolishing protein-binding capacity. The presence of serum albumin reduces cell stress and the high salt wash step allows stringent purification compared to using the His tag [18]. Moreover, the elution conditions with *d*-desthiobiotin are less harsh than for other systems, e.g., Ni-sepharose or protein G-sepharose. Generally, the expression levels vary dramatically between different extracellular matrix (ECM) proteins and receptor ectodomains. Not surprisingly, Ig-fold containing receptor ectodomains express at high levels. Even if we use proteins with the same folding domains, differences can be observed (Fig 1A). The N-terminal region of the laminin γ1 chain (γ1LN-LEa1-4, Fig 2) on its own showed high expression levels, whereas the homologous protein domain of netrin-1 (netrin-1 delta, Fig 2) was hardly expressed. Codon optimization of the *NTN1* cDNA only increased the expression levels marginally compared to that of laminin γ1 chain in the basic vector (data not shown).

In terms of eukaryotic expression, different expression tags have been previously described and fusion of receptor ectodomains to the Fc tag has been beneficial. Therefore, we tested different N-terminal truncated versions of the mFc tag to optimize netrin expression (S1B Fig). From the six tested variants, the version containing two cysteines in the hinge region revealed the highest expression level (data not shown). After additional codon optimization (S1C Fig), the expression level of the mFc was further increased about two fold (Fig 1B, mFc versus mFc-opt). Western blot analysis indicated that the mFc-opt tag alone showed the highest expression level compared to different polypeptides (Fig 1B and S1D Fig). Moreover, we also investigated the expression level of the isolated GST tag; however, almost no expression was observed (data not shown). Hence, the GST-tag was excluded from further analysis. To test the impact of different tag polypeptides on fusion protein expression levels, netrin-1 delta as well as netrin-4 delta (delta: missing the C-terminal NTR domain, S2 Fig) was fused to these tags. Surprisingly, the highest expression levels were observed not with the mFc-opt tagged fusion proteins but with the MBP-fused ones (Fig 1C and 1D). This result was further supported by testing several different recombinant proteins, e.g., the ectodomain (ecto) of DCC (deleted in colorectal cancer) fused to MBP (S3C Fig). Quantitative analysis revealed an increase in expression between 2 to 5 times compared to the expression level without MBP (S3A–S3C Fig). To investigate the general applicability of tethering recombinant proteins to the MBP tag in terms of protein production, we further tested intracellular (HSP90α and Gli1) as well as transmembrane (MMP14 and MEGF9) proteins fused to MBP. Strikingly, all proteins showed higher expression levels, albeit variation in their domain composition when fused to MBP compared to the basic vector system (Fig 2A and 2B). Furthermore, we tested the expression level of netrin-4 delta in different cell lines (COS-7 and CHO-K1), which are commonly used for protein expression. Notably, the MBP tag boosted fusion protein expression in all cell lines (Fig 2C). We therefore also tested the expression of mutated proteins, which are normally retained in the ER. Here, we chose the laminin β2 R246Q mutation (β2LN-LEa1-4), which causes congenital nephrotic syndrome. Transient expression of the wildtype versus the R246Q laminin β2 chain (S2 Fig) in HEK 293 cells revealed that MBP again enhanced protein synthesis and secretion. Without MBP no band was visible for the R246Q mutant, however upon fusion to MBP a strong signal was detected (Fig 2D).

**Fig 3:**
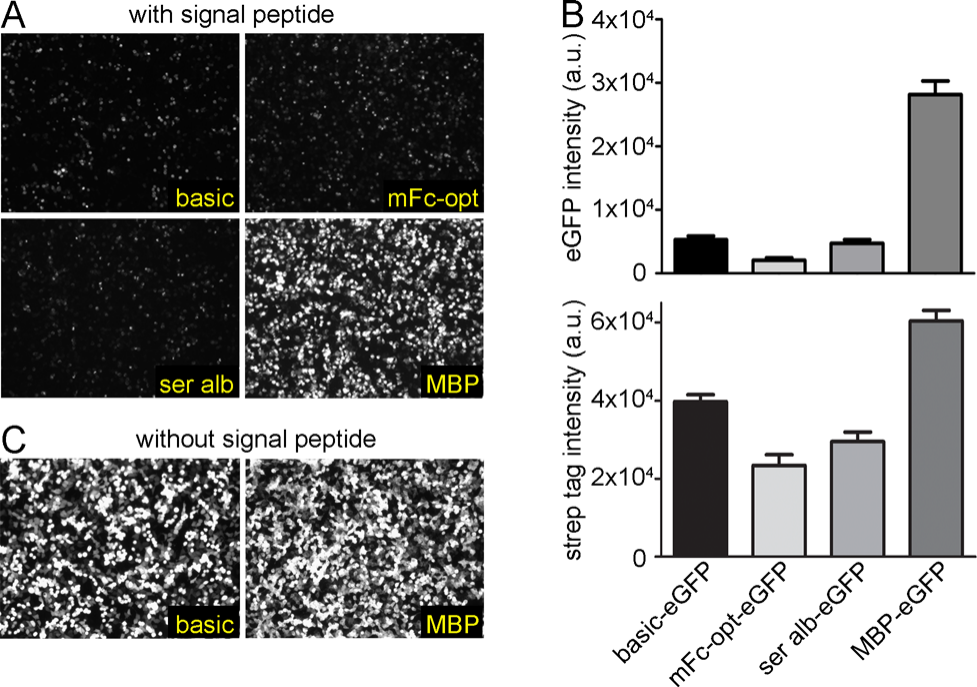
Enhanced expression of eGFP. HEK293 cells were transiently transfected with eGFP alone (basic) or fused to different expression tags. (A) All proteins contained a signal peptide sequence for secretion. The eGFP signals were visualized by fluorescence microscopy. (B) To quantify the secreted eGFP, the supernatants from the HEK293 cells were excited with a laser at 488 nm and the emission signal was detected at 509 nm (top). Densitometric analysis of secreted eGFP from HEK293 cells by western blot analysis using a Strep-Tactin®-HRP conjugate to detect Strep II tagged eGFP fusion proteins (bottom). (C) Without signal peptide, eGFP was transiently expressed intracellularly with or without fused MBP. eGFP signals were visualized by fluorescence microscopy. (a.u.: arbitrary unit).

**Fig 4:**
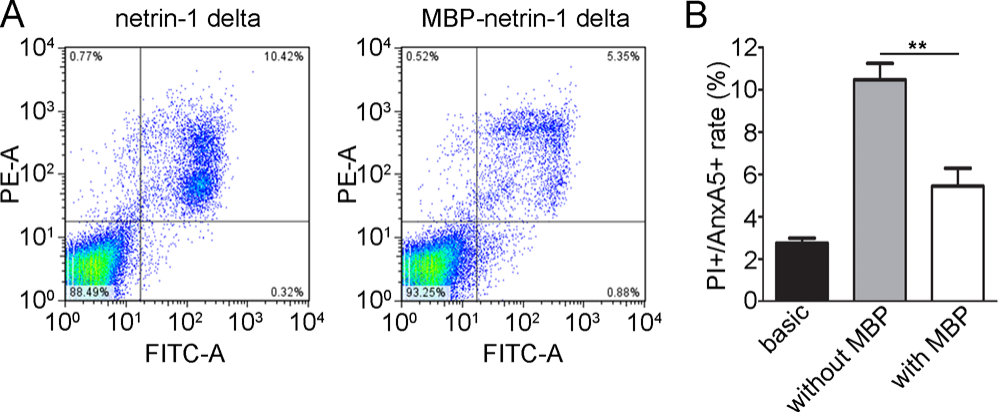
Analysis of apoptosis upon transient transfection of netrin-1 delta and netrin-1 delta tethered to MBP. (A) Apoptosis analysis by PI and annexin A5 co-staining. FACS analysis of transient transfected HEK293 cells with either netrin-4 delta or MBP-netrin-4 delta. (B) Graph shows the proportion of PI+/AnxA5+ cells in cells transfected with the empty basic vector, netrin-1 delta in the basic as well as the MBP vector (mean ± s.d.; *n* = 3; **P < 0.01). *P* values, Student’s *t*-test.

To investigate if MBP tethering affects the secretory pathway, we tested the secretion of the green fluorescent protein (eGFP) fused to different tags (Fig 3A). Fluorescence microscopy analysis suggested that indeed the eGFP protein expression was also enhanced upon fusion to MBP. Interestingly, whereas western blot analysis revealed a moderate increase in protein secretion of the MBP-eGFP compared to eGFP alone (Fig 3B, bottom), the difference was much more prominent for the measured eGFP signal (Fig 3B, top). This might indicate that MBP enhances or stabilizes the eGFP protein folding. To verify that this is due to the secretion through the ER and Golgi apparatus, we expressed both variants without the signal peptide. As expected, a stronger signal was detected for MBP-eGFP fusion protein compared to eGFP without MBP (Fig 3C). We further investigated the influence of MBP on apoptosis upon transient transfection. After transfection of HEK293 cells with netrin-1 delta fused to MBP (MBP-netrin-1 delta), we observed a significant decrease of about 50% in number of dead cells (PI+/Anx5V+) compared to netrin-1 delta alone (Fig 4A (representative FACS plot) and 4B (quantitative analysis)).

Since MBP expressed in HEK293 cells has not been studied in detail, we investigated recombinant MBP and its fusion proteins biochemically. First, we studied the glycosylation pattern, which revealed absence of N-glycosylation in MBP (S4A Fig). However, we detected O-glycosylation on sites within the GluC-fragment SWSHPQFE of the Strep II tag, which has not been reported previously (S4B Fig). Since we noticed a significant increase in protein secretion of MBP-fused proteins, we investigated whether MBP might influence the glycosylation pattern of the accompanied proteins. We compared the glycosylation of netrin-4 delta fused to MBP (MBP-netrin-4 delta) versus netrin-4 delta in the basic vector (S4C and S4D Fig). Our glycosylation analysis indicated slightly more glycans with shorter peripheral chains and an additional unprocessed N-glycan core structure of 1117.5 Da in MBP-netrin-4 delta.

**Fig 5:**
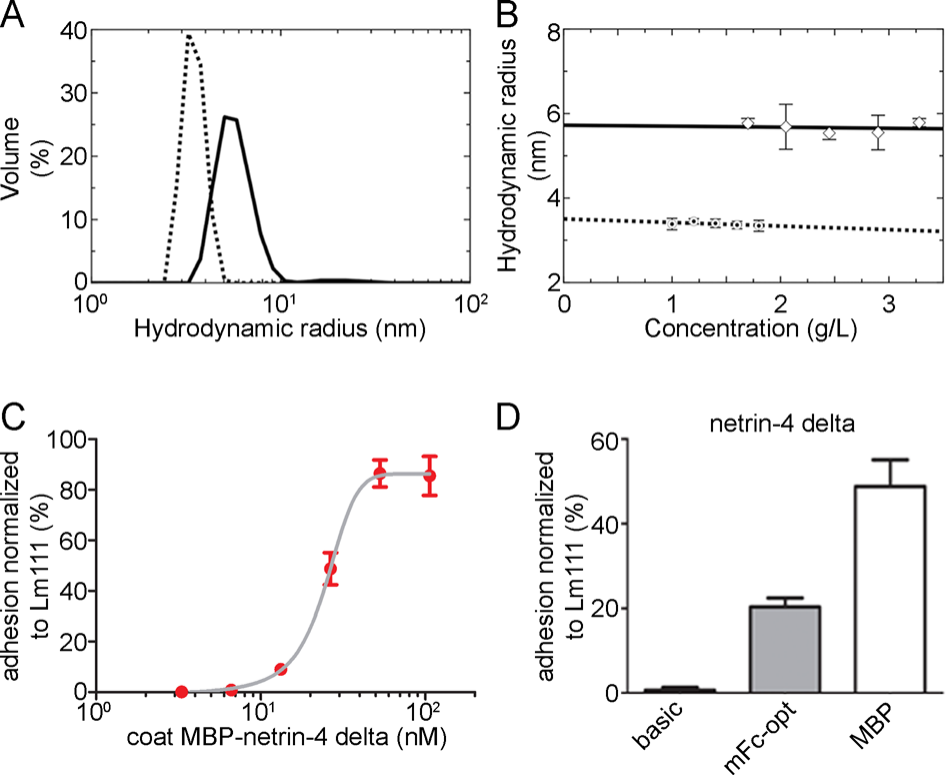
Biophysical analysis of MBP as well as MBP fusion proteins and cell attachment studies. (A) Light scattering profile obtained for a 1.80 mg/mL solution of MBP (dotted line) and for a 3.28 mg/mL solution of MBP-netrin-4 delta (solid line) in the TBS buffer display the respective hydrodynamic radius. (B) Concentration dependence of the hydrodynamic radius of MBP (dotted line) and MBP-netrin-4 delta (solid line), respectively, deduced from the peaks of DLS profiles. (C-D) B16-F1 cells were allowed to adhere to surface coated with MBP-netrin-4 delta. (C) Dependence of cell attachment on the coating concentration of MBP-netrin-4 delta. (D) Cell attachment at E_50_ coating concentration (MBP-netrin-4 delta) for different netrin-4 delta proteins (with and without tag).

Moreover, we analyzed the tendency for oligomerization of the MBP tag and MBP-netrin-4 delta. Dynamic light scattering (DLS) analysis revealed that although MBP was purified using only affinity chromatography, the preparation was highly monodisperse and aggregation free. The DLS measurements at multiple concentrations suggested that MBP behaves as a stable monomer in solution (Fig 5A and 5B). Similarly, DLS studies also suggested that the MBP-netrin-4 delta fusion protein was highly pure and behaves as a stable monomer in solution at multiple concentrations. To further enhance the expression of MBP, codon optimization was performed. However in this case, codon optimization did not increase the expression, in contrast to what was demonstrated previously for the Fc sequence (data not shown). To address the question whether the MBP fold alone is sufficient for the observed enhancing effect, we tested related MBP proteins from other organisms, e.g., *Pyrococcus furiosus* (*P*. *furious*) and *Pyrobaculum aerophilum* (*P*. *aerophilum*), as expression tags. Even though these two MBP related proteins show only a 28% sequence homology to MBP (S5 and S6 Figs), the expression levels of the respective fusion proteins were equal to MBP from *E*. *coli* (data not shown) indicating that the overall folding structure is sufficient for the observed positive effect.

The Fc tag is widely used for biochemical studies such as ELISA due to its preference of being absorbed to the plastic surface, which guarantees directional coating. Therefore, we performed cell attachment studies with B16-F1 mouse melanoma cells to netrin-4 delta fused to Fc or MBP versus without a tag. Netrin-4 delta without a tag only slightly mediated cell attachment. This might be due to a preferential absorption of the cell adhesion surface within netrin-4 to the plastic. Strikingly, after fusing MBP to netrin-4 delta, we observed a concentration dependent binding curve (Fig 5C). These cells did not bind to MBP or to mFc-opt alone (not shown). Interestingly, B16-F1 cells showed an improved binding to MBP-netrin-4 delta compared to mFc-opt-netrin-4 delta (Fig 5D).

## Discussion

For a number of applications in life sciences and biomedicine such as crystallization and therapeutic studies, large quantities of recombinant proteins are required. Therefore, there is a necessity of optimizing protein expression in eukaryotic cells. In recent years, transient transfection of HEK293 or CHO cells has become the gold standard for protein production [19]; however, it is a very expensive approach for obtaining high amounts of purified proteins due to cost intensive plasmid isolations and transfection reagents. In our laboratory we attempted to integrate various approaches to achieve higher levels of protein expression in mammalian cells [20]. However, we could not see any benefits in comparison to the classical episomal pCEP-4 vector (data not shown). Furthermore, integrating DNA insulation fragments and/or the woodchuck hepatitis virus post-transcriptional regulatory element (WPRE) into the episomal pCEP-4 vector had only slight effects on the expression levels of recombinant proteins upon transient transfection (data not shown). In particular, the expression level of the ECM protein netrin-1 delta in our basic vector (Fig 1C) was very low. Therefore, we pursued the approach to fuse netrin-1 to different polypeptides. Remarkably, joining netrin-1 delta or other extracellular, intracellular, and transmembrane proteins with the bacterial MBP protein strongly improved their expression levels. None of the other tested expression tags (Fc, GST, SlyD, serum albumin, and FKBP12 + IF) showed similar enhancement. This was rather surprising since some of the tested bacterial proteins also possess isomerase and or chaperone activity [8]. Additionally, we also tested different bacterial as well as eukaryotic isomerases, e.g., human peptidylprolyl isomerase A or B, bacterial peptidyl-prolyl cis-trans isomerase A, and dsba from *E*. *coli*. but without any success.

Different studies reported that some amino acids are crucial for the chaperone activity but not for the protein expression level of MBP in bacteria. Three mutations near one end of the maltose-binding cleft reduced the solubility of fusion proteins [5], indicating that this hydrophobic maltose binding pocket plays a role in keeping aggregation-prone folding intermediates in solution by preventing self-association. However, a comparison of the three MBP proteins from different bacteria tested here reveals that these amino acids are not conserved (S5 Fig), although all of them show similar enhanced expression of recombinant fusion proteins in mammalian cell. To substantiate the finding that this pocket does not play a role in our observed expression enhancing activity of MBP, one would have to repeat the experiments of Fox and colleagues [5] in mammalian cells and perform a new mutational amino acid screen. The benefit of using MBP fusion proteins is further supported by previous work in which thermally stable MBP from *P*. *furiosus* was fused to a single domain antibody [21]. This fusion protein retained its activity even after heat treatment to 70°C for 1 h [21].

Our results clearly indicate that protein expression levels of extracellular, intracellular as well as transmembrane proteins tethered to MBP are strongly enhanced in mammalian cells, however we have no clear explanation for this observation. In case of secreted proteins, the effect might be tied to the secretory pathway, whereby MBP might accelerate folding and/or retaining time of the MBP fusion protein within the ER or Golgi apparatus. However, since we also see an effect on intracellular proteins (eGFP without a signal peptide) MBP could lead to a faster mRNA turnover for translation. Additionally, MBP fusion leads to a reduced number of dying cells after transfection of the expression vector.

In summary, the utilization of the MBP tag in eukaryotic expression systems is a novel and beneficial approach for expression of recombinant proteins. MBP fusion proteins cause less cell death during transfection and facilitate enhanced secretion through the ER and Golgi apparatus. However, the exact underlying mechanism remains elusive and has to be addressed in the future. MBP enables the production, secretion, and purification of netrin-1 subdomains [22], intracellular as well as transmembrane proteins and mutant ECM proteins, such as the congenital nephrotic syndrome causing laminin β2 R246Q mutated chain [23]. MBP was recently used to express a TRPA1 ion channel as a MPB fusion protein in mammalian cells to perform single-particle electron cryo-microscopy [24]. However, the authors did not mention the rationale of using the MBP tag. A plausible reason could be that the MBP protein was used as a purification tag since the protein can be released from the amylose resin under mild conditions. However, our use of the Strep II tag in combination with the MBP tag is beneficial for protein purification since the Strep II tag is less likely to be affected by the protein to which it is fused. Furthermore, we could demonstrate that MBP fusion proteins can be used for cell adhesion studies due to the preference of MBP to absorb to plastic surfaces similar to the Fc tag. However, the MBP tag reveals a strong advantage compared to the Fc tag because it does not affect or induce artificial dimerization as the Fc tag does. In the near future, we will compare episomal protein expression systems to recently developed methods to further optimize recombinant protein production. For example, since we often observed a down regulation of protein expression after selection of HEK293 cells with puromycin, a stable integration into the genome might be beneficial, e.g., retroviral or transposase based transfection systems [25, 26].

## Supporting Information

S1 Fig Overview of the constructs used in this study. Domain overview of the laminin and netrin protein versions used in this study.
(TIF)

S2 Fig Sequence and construct informations. (A) Amino acid sequence of the N-terminal sequence of the construct is composed of the signal peptide (bold) followed by the double Strep II tag (underlined). The tandem tag allows the direct purification of recombinant proteins from serum containing cell supernatants. (B) Different N-terminal versions of the mouse Fc tag were cloned and tested. Codon optimized version F produced the highest expression. (C) DNA sequence of the codon optimized mFc-opt. (D) Schematic drawings of the PCEP vectors generated for this study. (BM40: osteonectin signal peptide sequence; 2xStrep II, double Strep II tag; thr: thrombin cleavage sequence; MBP: maltose binding protein; mFc opt: codon optimized Fc part from the mouse IgG protein; ser alb: serum albumin; isom.: modified SlyD; MCS: multi cloning site).
(TIF)

S3 Fig Protein quantification of transiently transfected HEK293. (A) Netrin-4 delta without (basic), with a serum albumin tag (ser alb) or a MBP tag were transiently expressed in HEK293 and analysed via western blot analysis. The graph on the right represents the densiometric analysis. (B and C) Quantification of the expression level of cells transfected with netrin-1 delta (B) and DCC ecto (C) with or without the MBP tag. (a.u.: arbitrary unit)
(TIF)

S4 Fig Glycan analysis. MALDI-MS spectrum of permethylated N-glycans derived by PNGase F digestion of recombinantly expressed MBP (A), netrin-4 delta (C), and MBP-netrin-4 delta (D). All glycans are detected as sodium adducts and show an additional signal at -14 Da (-Me, indicated by dashed lines). Most glycans are detected as -54 Da (-NaOCH_3_). (A) No glycans were detected in MBP tag alone. (C, D) Slightly more glycans with shorter peripheral chains and an additional unprocessed N-glycan core structure at 1117.5 Da were observed in MBP-netrin-4 delta (D) than in netrin-4 alone (C). The signal observed at 1121 Da in (C) is a contamination. (B) MBP by ESI-MS/MS of m/z 1164.4 corresponding to the GluC-fragment SWSHPQFE (1017.4 Da) of the StrepII Tag modified with H2N2S (2039.6 Da). A fragmentation of the peptide backbone is not observed due to the preferred glycan fragmentation. However, since the protein was highly purified and no other peptides with the observed mass exist, there is no doubt about the identity of the peptide and that this peptide has to be O-glycosylated. (H: hexose, N: N-acetylhexosamine, S: sialic acid, F: fucose).
(TIF)

S5 Fig Sequence alignment of different maltose-binding periplasmic proteins (MBPs). Amino acid sequences were aligned using ClustalW2 Multiple sequence alignment.
(TIF)

S6 Fig Structural comparison. The MBP structures of *E*. *Coli* (4MBP) and *P*. *furiosus* (1ELJ) were previously solved. *P*. *aerophilum* MBP protein has 52% sequence identity and about 78% sequence similarity to *E*. *Coli* MBP. The structure coordinates of *P*. *furiosus* was used to generate a 3D modell of *P*. *aerophilum*. (blue: basic, red: acidic amino acid).
(TIF)
