# Supplementary figures and images for: Maltose-Binding Protein (MBP), a Secretion-Enhancing Tag for Mammalian Protein Expression Systems
Source: PLoS One. 2016 Mar 30;11(3):e0152386. doi: 10.1371/journal.pone.0152386 (PMC4814134; doi:10.1371/journal.pone.0152386)

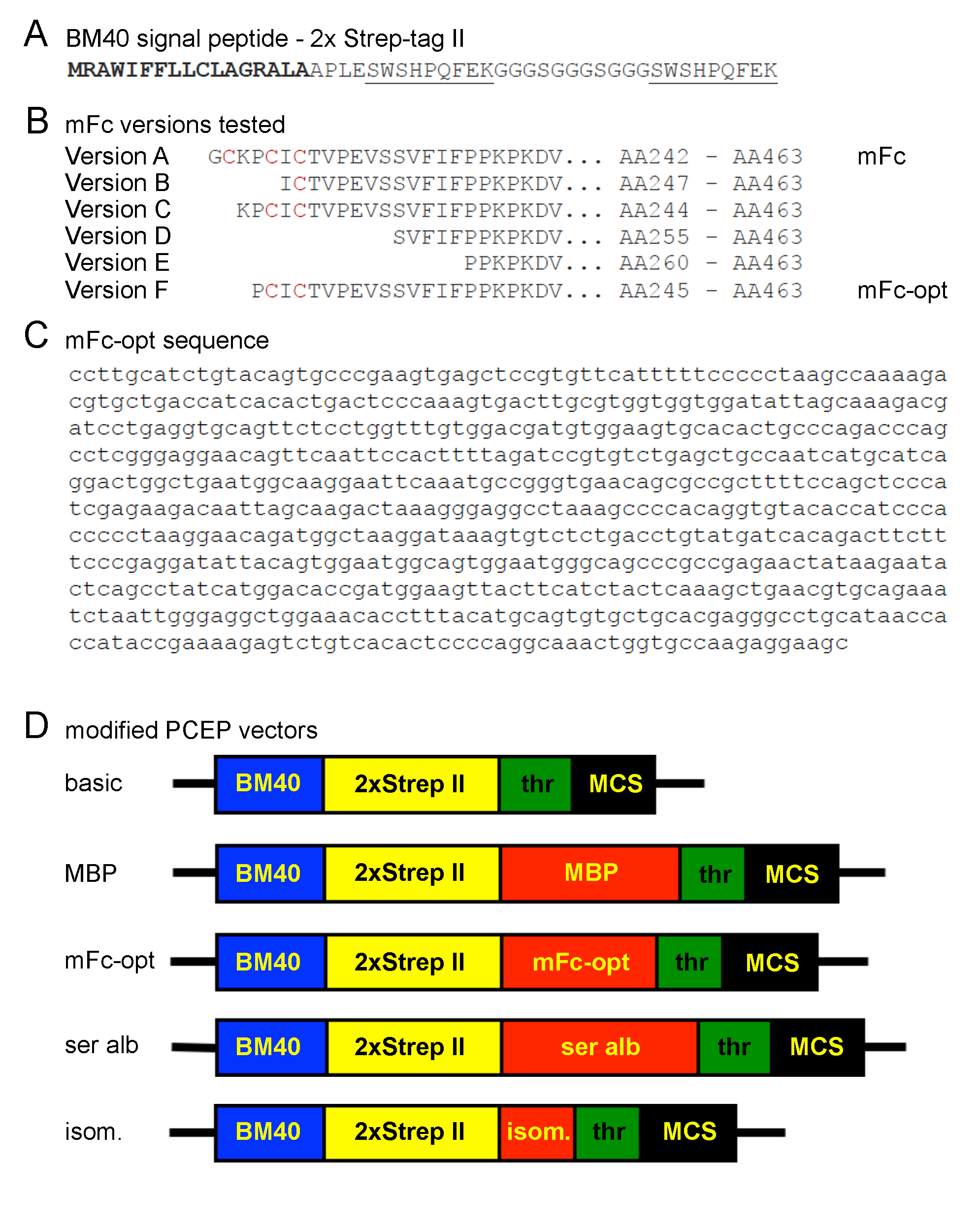

Supplement: S1 Fig — Domain overview of the laminin and netrin protein versions used in this study. (TIF) [file pone.0152386.s001.tif]

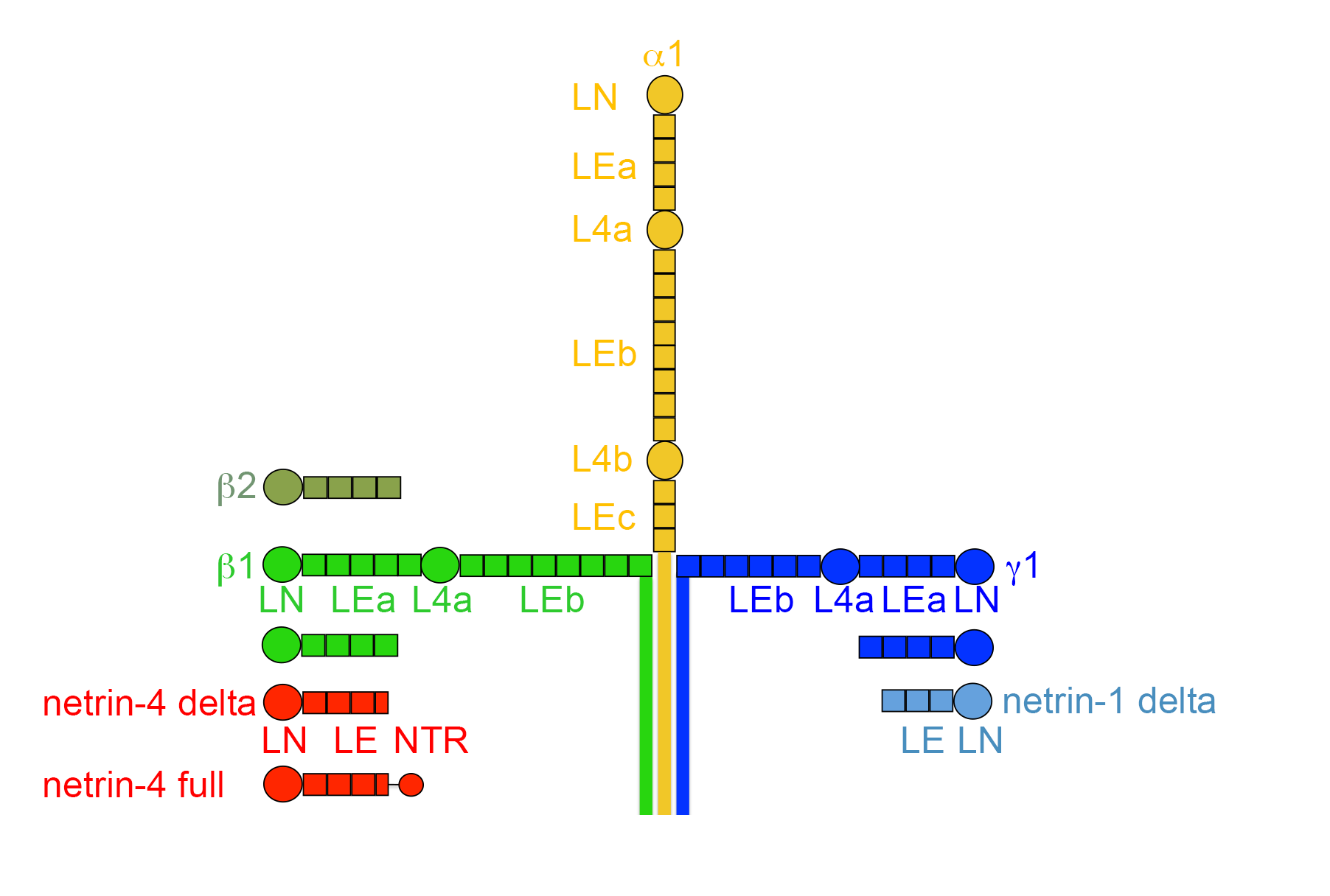

Supplement: S2 Fig — (A) Amino acid sequence of the N-terminal sequence of the construct is composed of the signal peptide (bold) followed by the double Strep II tag (underlined). The tandem tag allows the direct purification of recombinant proteins from serum containing cell supernatants. (B) Different N-terminal versions of the mouse Fc tag were cloned and tested. Codon optimized version F produced the highest expression. (C) DNA sequence of the codon optimized mFc-opt. (D) Schematic drawings of the PCEP vectors generated for this study. (BM40: osteonectin signal peptide sequence; 2xStrep II, double Strep II tag; thr: thrombin cleavage sequence; MBP: maltose binding protein; mFc opt: codon optimized Fc part from the mouse IgG protein; ser alb: serum albumin; isom.: modified SlyD; MCS: multi cloning site). (TIF) [file pone.0152386.s002.tif]

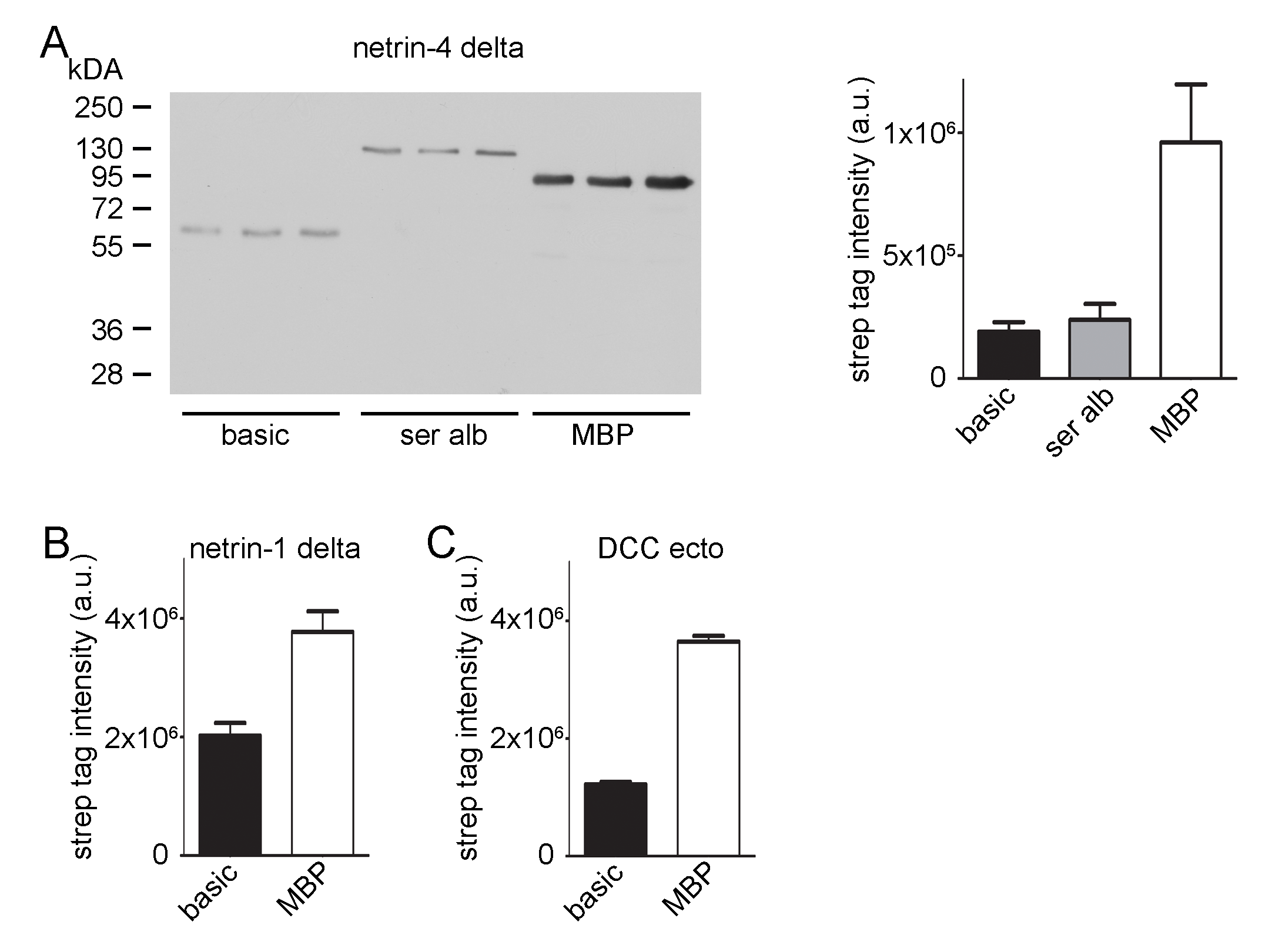

Supplement: S3 Fig — (A) Netrin-4 delta without (basic), with a serum albumin tag (ser alb) or a MBP tag were transiently expressed in HEK293 and analysed via western blot analysis. The graph on the right represents the densiometric analysis. (B and C) Quantification of the expression level of cells transfected with netrin-1 delta (B) and DCC ecto (C) with or without the MBP tag. (a.u.: arbitrary unit) (TIF) [file pone.0152386.s003.tif]

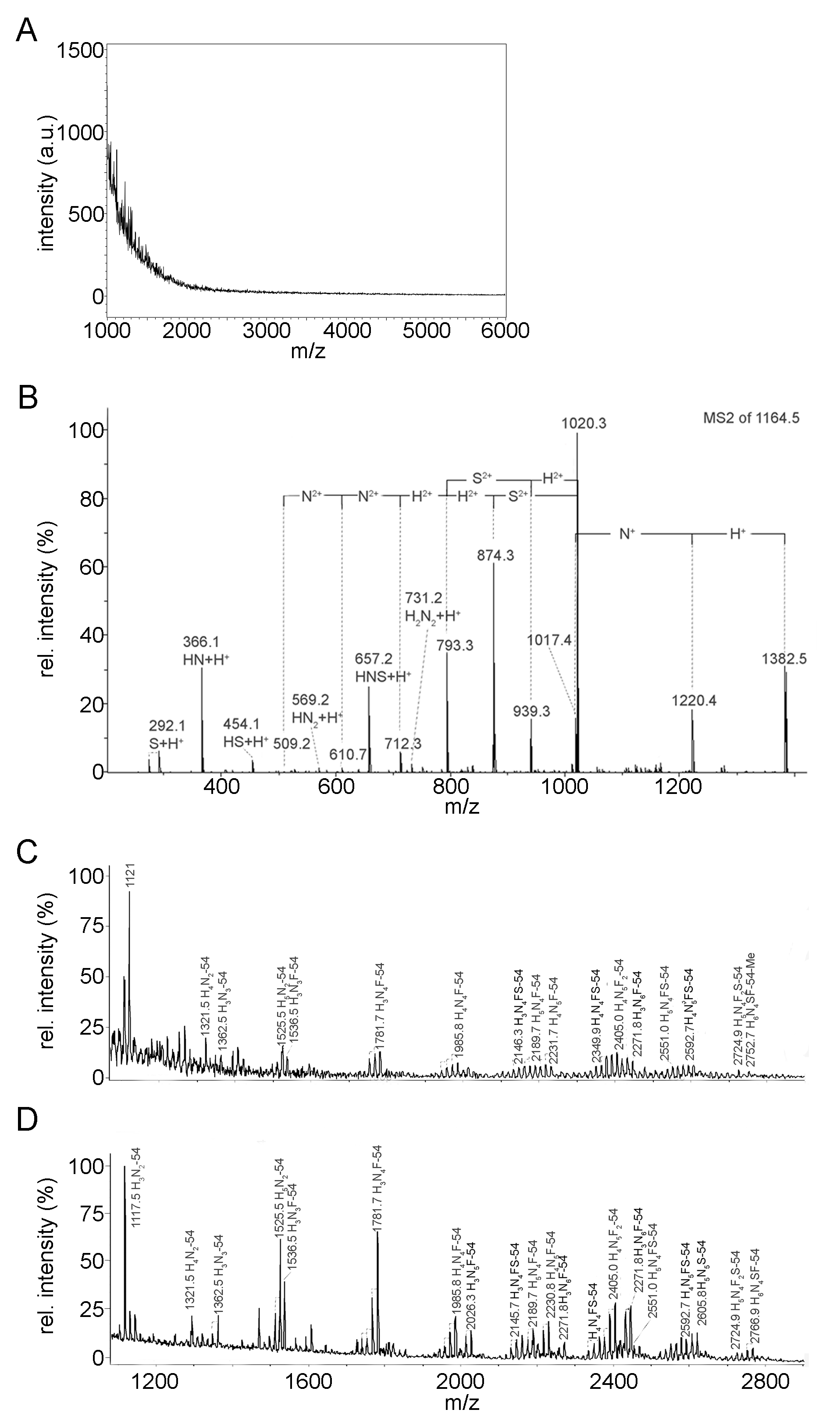

Supplement: S4 Fig — MALDI-MS spectrum of permethylated N-glycans derived by PNGase F digestion of recombinantly expressed MBP (A), netrin-4 delta (C), and MBP-netrin-4 delta (D). All glycans are detected as sodium adducts and show an additional signal at -14 Da (-Me, indicated by dashed lines). Most glycans are detected as -54 Da (-NaOCH3). (A) No glycans were detected in MBP tag alone. (C, D) Slightly more glycans with shorter peripheral chains and an additional unprocessed N-glycan core structure at 1117.5 Da were observed in MBP-netrin-4 delta (D) than in netrin-4 alone (C). The signal observed at 1121 Da in (C) is a contamination. (B) MBP by ESI-MS/MS of m/z 1164.4 corresponding to the GluC-fragment SWSHPQFE (1017.4 Da) of the StrepII Tag modified with H2N2S (2039.6 Da). A fragmentation of the peptide backbone is not observed due to the preferred glycan fragmentation. However, since the protein was highly purified and no other peptides with the observed mass exist, there is no doubt about the identity of the peptide and that this peptide has to be O-glycosylated. (H: hexose, N: N-acetylhexosamine, S: sialic acid, F: fucose). (TIF) [file pone.0152386.s004.tif]

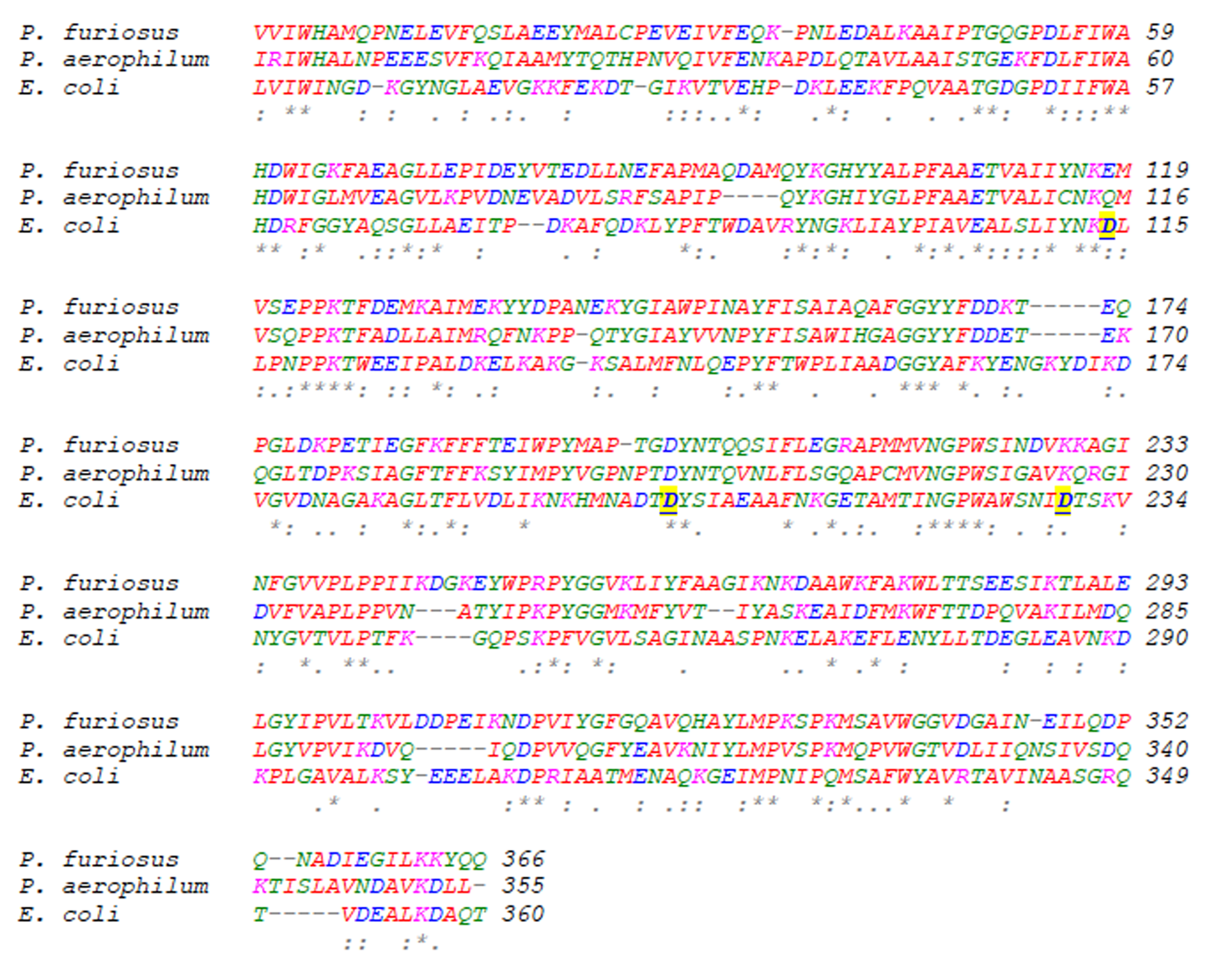

Supplement: S5 Fig — Amino acid sequences were aligned using ClustalW2 Multiple sequence alignment. (TIF) [file pone.0152386.s005.tif]

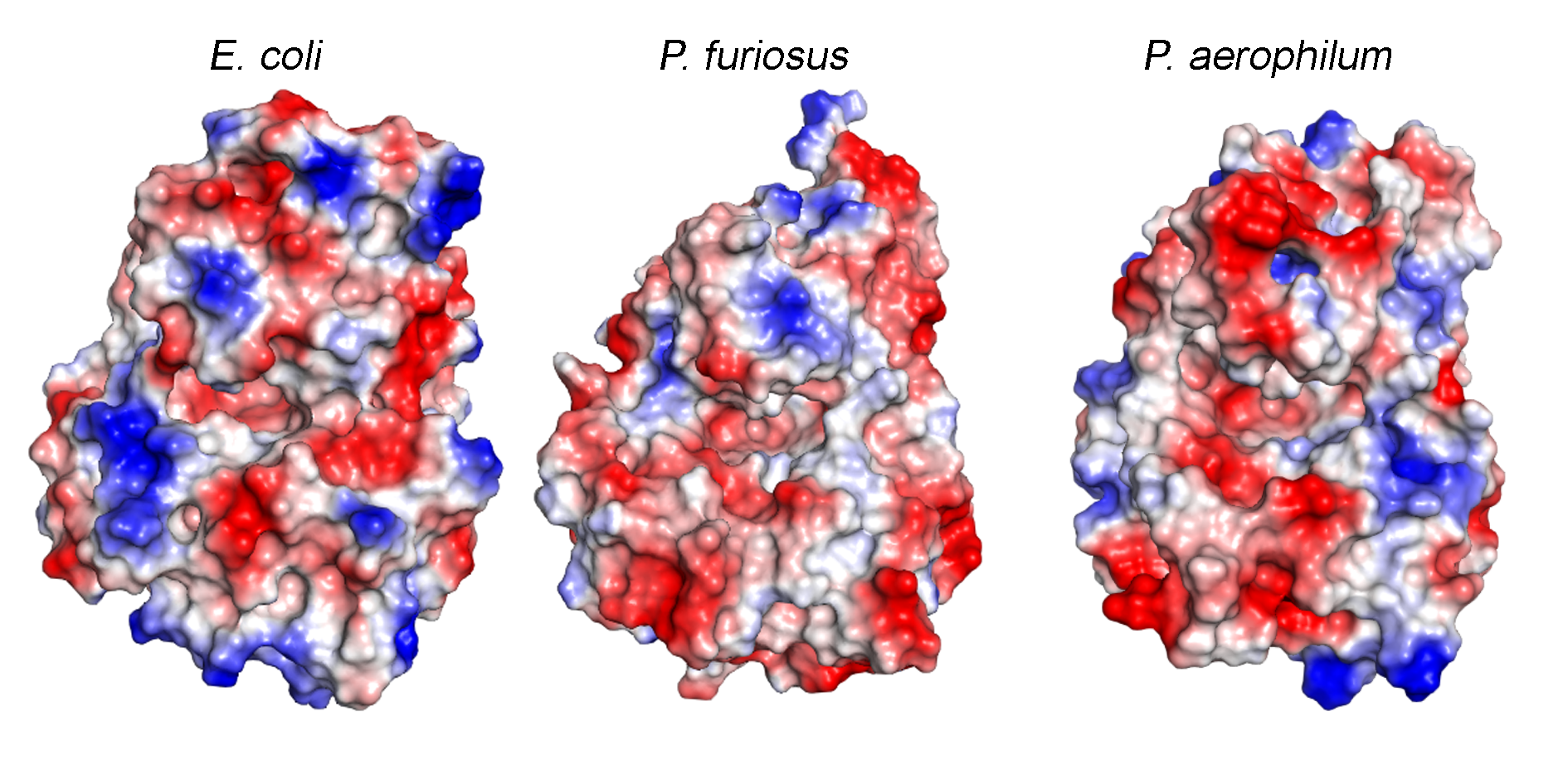

Supplement: S6 Fig — The MBP structures of E. Coli (4MBP) and P. furiosus (1ELJ) were previously solved. P. aerophilum MBP protein has 52% sequence identity and about 78% sequence similarity to E. Coli MBP. The structure coordinates of P. furiosus was used to generate a 3D modell of P. aerophilum. (blue: basic, red: acidic amino acid). (TIF) [file pone.0152386.s006.tif]
